# Comparative evaluation of different modalities for measuring *in vivo* carnosine levels

**DOI:** 10.1371/journal.pone.0299872

**Published:** 2024-03-27

**Authors:** Alok R. Amraotkar, David Hoetker, Mohammad J. Negahdar, Chin K. Ng, Pawel Lorkiewicz, Ugochukwu S. Owolabi, Shahid P. Baba, Aruni Bhatnagar, Timothy E. O’Toole

**Affiliations:** 1 Christina Lee Brown Envirome Institute, University of Louisville, Louisville, KY, United States of America; 2 Division of Environmental Medicine, Department of Medicine, University of Louisville, Louisville, KY, United States of America; 3 Department of Radiology, University of Louisville, Louisville, KY, United States of America; 4 Department of Chemistry, University of Louisville, Louisville, KY, United States of America; University of Houston, UNITED STATES

## Abstract

Carnosine is an endogenous di-peptide (β-alanine -L- histidine) involved in maintaining tissue homeostasis. It is most abundant in skeletal muscle where its concentration has been determined in biopsy samples using tandem mass spectrometry (MS-MS). Carnosine levels can also be assessed in intact leg muscles by proton magnetic resonance spectroscopy (1H-MRS) or in blood and urine samples using mass spectrometry. Nevertheless, it remains uncertain how carnosine levels from these distinct compartments are correlated with each other when measured in the same individual. Furthermore, it is unclear which measurement modality might be most suitable for large-scale clinical studies. Hence, in 31 healthy volunteers, we assessed carnosine levels in skeletal muscle, via 1H-MRS, and in erythrocytes and urine by MS-MS. While muscle carnosine levels were higher in males (C2 peak, p = 0.010; C4 peak, p = 0.018), there was no sex-associated difference in urinary (p = 0.433) or erythrocyte (p = 0.858) levels. In a linear regression model adjusted for age, sex, race, and diet, there was a positive association between erythrocyte and urinary carnosine. However, no association was observed between 1H-MRS and erythrocytes or urinary measures. In the relationship between muscle versus urinary and erythrocyte measures, females had a positive association, while males did not show any association. We also found that 1H-MRS measures were highly sensitive to location of measurement. Thus, it is uncertain whether 1H-MRS can accurately and reliably predict endogenous carnosine levels. In contrast, urinary and erythrocyte carnosine measures may be stable and in greater synchrony, and given financial and logistical concerns, may be a feasible alternative for large-scale clinical studies.

## Introduction

Carnosine is a naturally-occurring histidyl dipeptide (β-alanine-L-histidine) found in abundance in skeletal muscle, brain, and heart [1, 2]. Because of its unique chemistry, carnosine can buffer intracellular pH, chelate transition metals, and form conjugates with unsaturated aldehydes such as acrolein. Thus, carnosine is believed to play an important physiological role in maintaining tissue homeostasis. Indeed both carnosine, as well as its precursor amino acid β-alanine, have found wide-spread use as over-the-counter dietary supplements for the enhancement of athletic performance [3–5]. Related analogs of carnosine include methylated variants (anserine: β-alanine-N1-methyl-histidine; balenine: β-alanine-N3-methyl-histidine), acetylated variants (N-acetylcarnosine), and those with histidine substitutions (homocarnosine, carcinine) [2]. While some of these variants may have species- or tissue-specific expression patterns [2, 6], it is believed, as with carnosine, that they have protective roles in highly metabolic tissues.

In addition to stabilizing metabolism, carnosine also has anti-oxidant and anti-inflammatory properties, and efficacy of carnosine in protecting from cardiovascular and neurogenerative disorders has been examined in supplementation studies [7, 8]. Several animal or cell-based studies from our laboratory and others have shown that high levels of carnosine protect against tissue injury and dysfunction [9–13]. Specifically, we have found that dietary supplementation with carnosine decreases lesion formation in ApoE-null mice, reduces inflammation, improves wound healing responses, and protects the heart from ischemia reperfusion injury [10, 13–15]. We have also recently reported that carnosine supplementation improves stem cell function in murine models of air pollution exposure [16]. Carnosine supplementation also limited steatotic liver disease in a rat-disease model [17]. In human-based supplementation studies, carnosine appeared to improve glucose handling [18, 19], but had little effect on cardiac or vascular function in at risk individuals [20]. Carnosine supplementation, or administration of carnosine-rich foodstuffs has also shown promise in the treatment of cognitive dysfunction [21–23].

Given the potential therapeutic utility of exogenously delivered carnosine and it wide-ranging role in physiological homeostasis, quantitative measures of this di-peptide or its conjugates in different tissues and cellular compartments could be used as an indirect assessment of individual levels of oxidative stress, inflammation, and health risk [24, 25], as well as an index of the efficacy of supplementation. However many prior studies do little to evaluate this, nor is it clear how best to do this or what tissue source of carnosine to measure. Endogenous carnosine synthesis via carnosine synthetase predominantly occurs in skeletal muscle upon uptake of β-alanine and L-histidine from the bloodstream [26, 27]. Carnosine acquired by diet is not directly stored in skeletal muscles, but is first hydrolyzed in the bloodstream by carnosinase into β-alanine and L-histidine, which are eventually taken up by muscle tissue [28]. Most of bodily carnosine is stored in skeletal muscles [29] and it is frequently measured in muscle tissue biopsy samples using high-performance liquid chromatography tandem mass spectrometry (LC/MS/MS) [30]. However, as muscle biopsies are invasive and may cause localized trauma as well as increase the risk of infection or cause other complications, valid non-invasive alternatives are desirable. As an alternative, skeletal muscle carnosine levels can also be measured by proton magnetic resonance spectroscopy (1H-MRS) [31]. While 1H-MRS is a noninvasive approach [3, 32], the need for specialized equipment, trained personnel, and high cost of the procedure limits its widespread application in large clinical or research studies. Moreover, recent studies have raised concerns about the consistency of measuring carnosine via 1H-MR [33]. Therefore, more accessible and cost-effective approaches are needed for routine measurements of carnosine to replace or complement current invasive and imaging approaches.

Although carnosine is mostly stored in skeletal, it is also excreted in urine, where its levels can be accurately measured by high-performance LC/MS/MS [24, 25, 34]. Carnosine is also present in blood, but it is readily hydrolyzed into L-histidine and β-alanine by serum carnosinase, making it difficult to measure, even in the setting of chronic oral supplementation [35, 36]. Nevertheless, if successfully measured, blood carnosine measures might be better suited, as compared to muscle levels, for assessing prevention of lipoxidation [37], which has a strong association with endothelial dysfunction and cardiovascular disease (CVD) risk [38]. Recently it was reported that carnosine could be quantified in red blood cells (erythrocytes) [39]. Importantly, carnosine in erythrocytes is protected from serum carnosinase degradation [40]. In contrast to its varying levels in plasma due to hydrolysis, carnosine in erythrocytes may represent a more stable pool, relatively insensitive to fluctuations resulting from tissue absorption and/or hydrolysis. Moreover, because blood is a non-localized tissue that is involved in metabolic activity throughout the body, measurements of carnosine in erythrocytes may be a reliable indicator of systemic carnosine abundance.

In the present study, we simultaneously measured carnosine levels in urine, blood, and skeletal muscle of the same individuals, to determine whether its levels in different cellular compartments are correlated. Thereby, we attempt to understand how *in-vivo* carnosine stores in individual tissues correlate with each other, and whether carnosine measurements in erythrocytes could be used as a readily available source for larger clinical studies.

## Materials and methods

### Study design and cohort

Following Institutional Review Board (IRB) approval, healthy participants of either sex and any race, 18 years of age and above, were invited to participate in the study. The primary outcome measures of this study were *in-vivo* levels of carnosine as measured in erythrocytes, urine, and skeletal muscle (1H-MRS). Therefore only those who agreed to undergo all three study procedures: venous blood draw, urine sample collection, and a 1H-MRS scan were enrolled. Participants were excluded if they had a contraindication for the 1H-MRS procedure which includes but is not limited to claustrophobia, cardiac pacemakers, defibrillator, cochlear implants, tissue expander, aneurysm clip, insulin pump, drug infusion pump, metallic foreign objects, metallic implants, orthopedic plates, or screws. All subjects provided signed informed consent and the study protocol conformed to the ethical guidelines of the 1975 Declaration of Helsinki. This study and all the protocols were approved by the Institutional Review Board of the University of Louisville (IRB # 18.0703). Participants involved in the study were recruited between 1/7/2018 and 11/5/2020.

### Specimen collection and processing

Participants arrived for the study in the mid-morning or early afternoon hours with no dietary or fasting limitations. Sample collection and processing were completed using standardized operating protocols. Blood samples were collected in a vacutainer tube (4mL), spray-coated with 75 USP units of lithium heparin (Becton Dickinson, Franklin Lakes, NJ). Tubes were gently inverted 6–8 times after blood draw and incubated in upright position on ice for 30 minutes before additional processing. Samples were then spun at 1000*xg* for 15 min to separate the plasma and cells. The plasma and buffy coat were drawn off, leaving a bed of packed erythrocytes. An aliquot of these cells (41μL) was added to 375μL of ice-cold water and used to determine protein concentration. An additional 41μl aliquot was added to 375μL of fixative solution (55% methanol, 45% water, 1μM Carnosine-d4 (CDN Isotopes). Both samples were mixed by vortexing and incubated on ice for 5 min. The fixed sample solution was then pipetted into an Amicon Ultra-0.5 Centrifugal Filter Unit (Merck KGaA, Darmstadt, Germany), centrifuged at 4°C 14000*xg* for 42 min and the filtrate was collected and stored at -80°C until analysis. Urine was collected in a sterile cup and incubated in upright position on ice for 30 min. Urine samples were then aliquoted and stored at -80°C until analysis. Collection of blood and urine samples was performed immediately after consent and prior to 1H-MRS measurement.

### Measurement of erythrocyte carnosine

The preparation of samples and quantitative analysis was done as previously described [39]. Filtrates from the lysed erythrocyte samples were thawed and diluted 10-fold with a solution of 75% acetonitrile:25% water. Analyte separation was achieved on a Waters ACQUITY UPLC H-Class System (BEH HILIC column equipped with an in-line frit filter unit) coupled with a Xevo TQ-S micro triple quadrupole mass spectrometer. The analytes were eluted using a binary solvent system consisting of 10 mM ammonium formate, 0.125% formic acid in 5% acetonitrile: 95% water for mobile phase A and 10 mM ammonium formate 0.125% formic acid in 95% acetonitrile: 5% water for mobile phase B at a flow rate of 0.55 mL/min. Initial conditions were 0.1: 99.9 A: B ramping to 99.9: 0.1 A:B over 5 min then quickly ramping to 0.1:99.9 A:B over 0.5 min. For carnosine quantification, m/z 227***→***110 and carnosine- d_4_ m/z 231***→***110 transition ions as well as two confirmation ions were followed. A standard curve was generated using serial dilutions of purified carnosine (Sigma) in the above-noted erythrocyte fixative solution. Levels of carnosine in the participant samples were quantified using the peak ratio of carnosine and carnosine-d_4_ internal standard, interpolated using the standard curve, and expressed as nmoles carnosine/mg protein.

The precision of this analytical method was validated by replicate analysis of samples with the highest and lowest concentrations. Human erythrocyte methanol extracts were pooled from the five lowest (150μL total) and three highest (90μL total) individual samples then aliquoted into three samples each. A frozen aliquot from each sample pool was thawed, diluted and analyzed each day by injecting the same sample ten times each day for three consecutive days. Relative variability was calculated from the coefficient of variation of replicates within one sample run and between different sample runs as described [41]. The combined inter- and intra-assay variability of the carnosine low pool was 18.67%, while that for the carnosine high pool was 8.08%. An estimate of the limit of quantitation (LoQ) was produced by using a carnosine standard curve under the assay conditions and found to be 30fmol carnosine injected on column while the limit of detection (LoD) was estimated to be 8fmol carnosine injected on column.

### Measurement of urinary carnosine

Urine samples were thawed and diluted 100-fold in a solution of 75% acetonitrile:25% water containing 30nM carnosine-d_4_ and tyrosine-histidine as internal standards. Samples were separated using a Waters ACQUITY UPLC H-Class System (BEH HILIC column equipped with an in-line frit filter unit) coupled with a Xevo TQ-S micro triple quadrupole mass spectrometer. The analytes were eluted using a binary solvent system consisting of 10 mM ammonium formate, 0.125% formic acid in 5% acetonitrile: 95% water for mobile phase A and 10 mM ammonium formate 0.125% formic acid in 95% acetonitrile: 5% water for mobile phase B at a flow rate of 0.55 mL/min. Initial conditions were 0.1: 99.9 A: B ramping to 99.9: 0.1 A:B over 5 min then quickly ramping to 0.1:99.9 A:B over 0.5 min. For carnosine quantification, m/z 227***→***110, and carnosine- d_4_ m/z 231***→***110 transition ions were followed. Confirmation transition ions for each molecule were followed as well. Creatinine levels were measured on an Ace Axcel Clinical Chemistry Analyzer (Alfa Wassermann). Final carnosine levels were quantified using the peak ratio of histidyl-dipeptide and carnosine-d_4_ internal standard, interpolated using a standard curve and expressed as values normalized to creatinine (nmoles/mg creatinine). The LoQ for urinary carnosine was estimated to be 23fmol while the LoD was estimated to be 6fmol applied.

### Proton magnetic resonance spectroscopy (1H-MRS)

**Fig 1:**
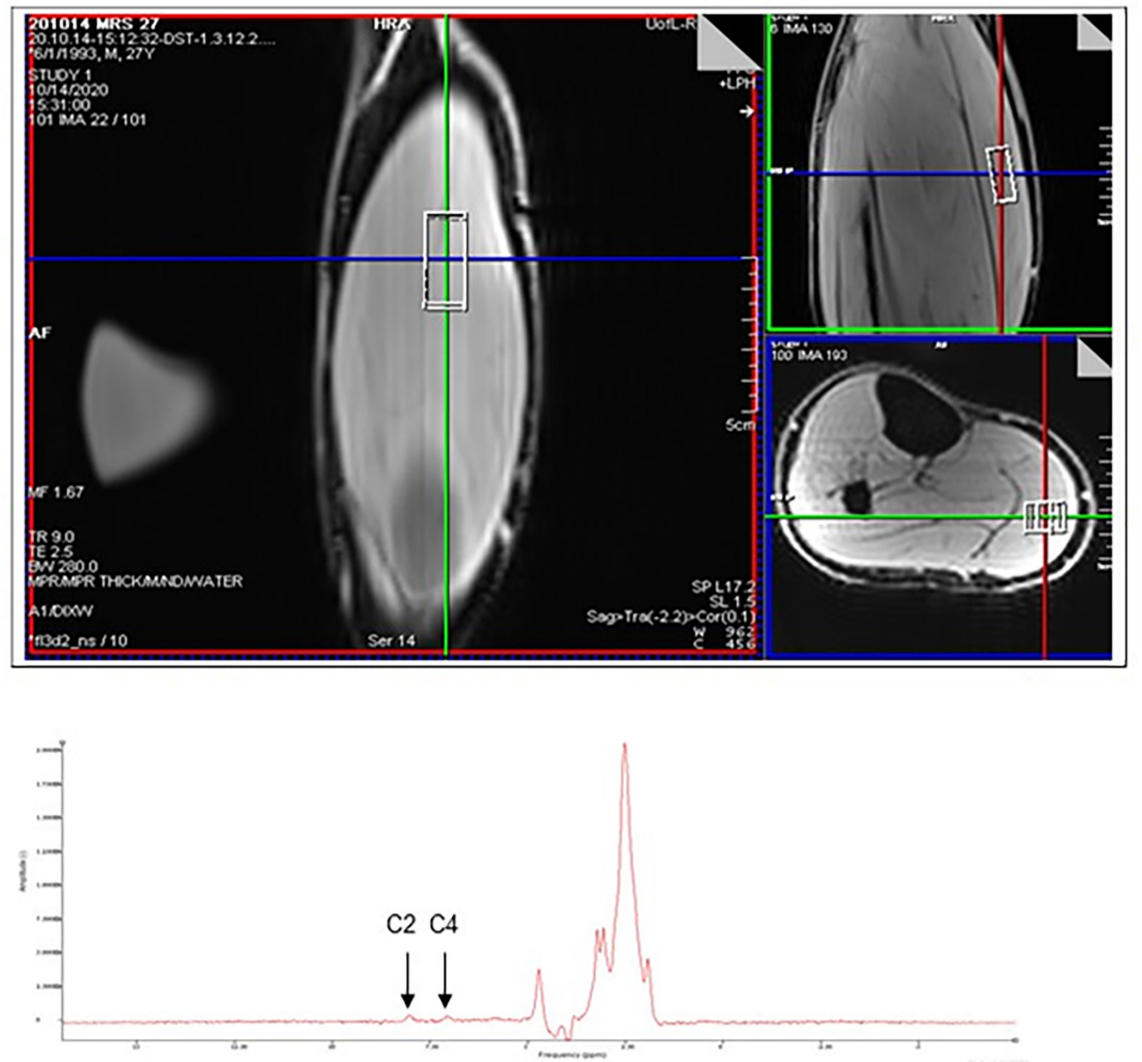
Magnetic resonance spectroscopy. Upper panel: Depicted are representative 1H-MRS images of the gastrocnemius. Boxes indicate voxel placement for the measurement of C2 and C4 peaks. Left; sagittal view; Right Top: coronal view; Right Bottom: axial view. Lower panel: Depicted is a representative MRS spectrum. Location of the carnosine C2 and C4 peaks is indicated.

1H-MRS data were acquired in a 3T clinical MRI scanner (Siemens Skyra, Syngo MR E11 software, Erlangen, Germany) following previously published procedures [32]. A flexible 18-channel RF surface body coil was wrapped around the leg of each volunteer for data acquisition. The right leg of study participants was secured in a cylindrical cavity enclosed with sandbags to minimize involuntary motion during data acquisition. The cylinder was positioned at the iso-center of the magnet to be in the most homogeneous region of the magnetic field. Single voxel spectroscopy spin echo sequence was utilized with the following optimized parameters: TR/TE = 1340 ms/ 69 ms; Voxel size: 10*12*30 mm (left-right, anterior-posterior, and foot-head directions respectively); number of signal averaging = 240; Flip angle: 70. Acquisition time for each voxel was 5 minutes and 32 seconds. The sampling voxel was positioned at the center of the gastrocnemius muscle and away from fat tissues as much as possible. Voxel location was confirmed via sagittal, coronal, and axial views (Fig 1). Manual shimming was utilized for each scan to optimize image quality.

Variance in carnosine measurement via 1H-MRS was assessed by performing serial measurements in a sub-group of study participants. Two distinct methods were tested: multiple measurements at single location and single measurement at multiple locations inside the gastrocnemius muscle as described above. For multiple measurements at single location, 3–5 measurements were acquired from the same anatomical location, without readjusting the voxel. For multiple locations, voxel was re-positioned to 3–5 suitable locations.

All MRS data were loaded into a spectrum analysis software package (jMRUI 5.0, Université Claude Bernard, Lyon, France) for carnosine peak quantification [42, 43]. An HLSVDPro filter was used in the time domain to suppress unwanted components of time signal after water peak suppression. The remaining graph was appraised after adjusting the phase. Carnosine is detected in two signals deriving from carbon 2 (C2) and carbon 4 (C4) of the imidazole ring, which resonate at 8 ppm and 7 ppm, respectively (Fig 1). All values were normalized to a water peak value in the identical voxel.

### Statistical analysis

Frequencies and percentages were reported for categorical variables. Shapiro-Wilk test was used to determine the normality of distribution. Since sex had the highest influence over the amount of skeletal muscle mass in an individual, we decided to compare the levels of these three depots between males and females. Raw values of non-normally distributed outcome measures (carnosine levels in three compartments) were compared between groups (males versus females) using Mann-Whitney U non-parametric test. The dependent outcomes, carnosine levels measured in each compartment (urine, erythrocytes, and 1H-MRS), were continuous variables and not normally distributed and hence were converted to natural log.

Coefficient of variance (CV) was calculated for both types of 1H-MRS repeat measurements: single location with multiple measurements and multiple locations with single measurements. CV was calculated as a percentage value using the following formula: (standard deviation / mean) * 100. Generalized Linear Models (GLM) were used to examine linear relationships between urine, erythrocytes, and 1H-MRS levels of carnosine. Carnosine levels between urine versus erythrocytes and urine versus muscle (C2 and C4 peaks of 1H-MRS) were analyzed in a model by adjusting for anticipated confounding effect of age, sex, race, diet, and creatinine. Carnosine levels between erythrocytes versus muscle (C2 and C4 peaks of 1H-MRS) were analyzed in a model by adjusting for anticipated confounding effect of age, sex, race, and diet. Creatinine was removed from this model since urine carnosine measures were not included.

To calculate the power for finding association between carnosine levels in different tissue depots, a two-tailed multiple regression fixed model with single regression coefficient was used wherein an alpha of 0.05 and an effect size of 0.35 SD units in a sample with 29 participants yielded 86% power.

## Results

### Cohort

**Fig 2:**
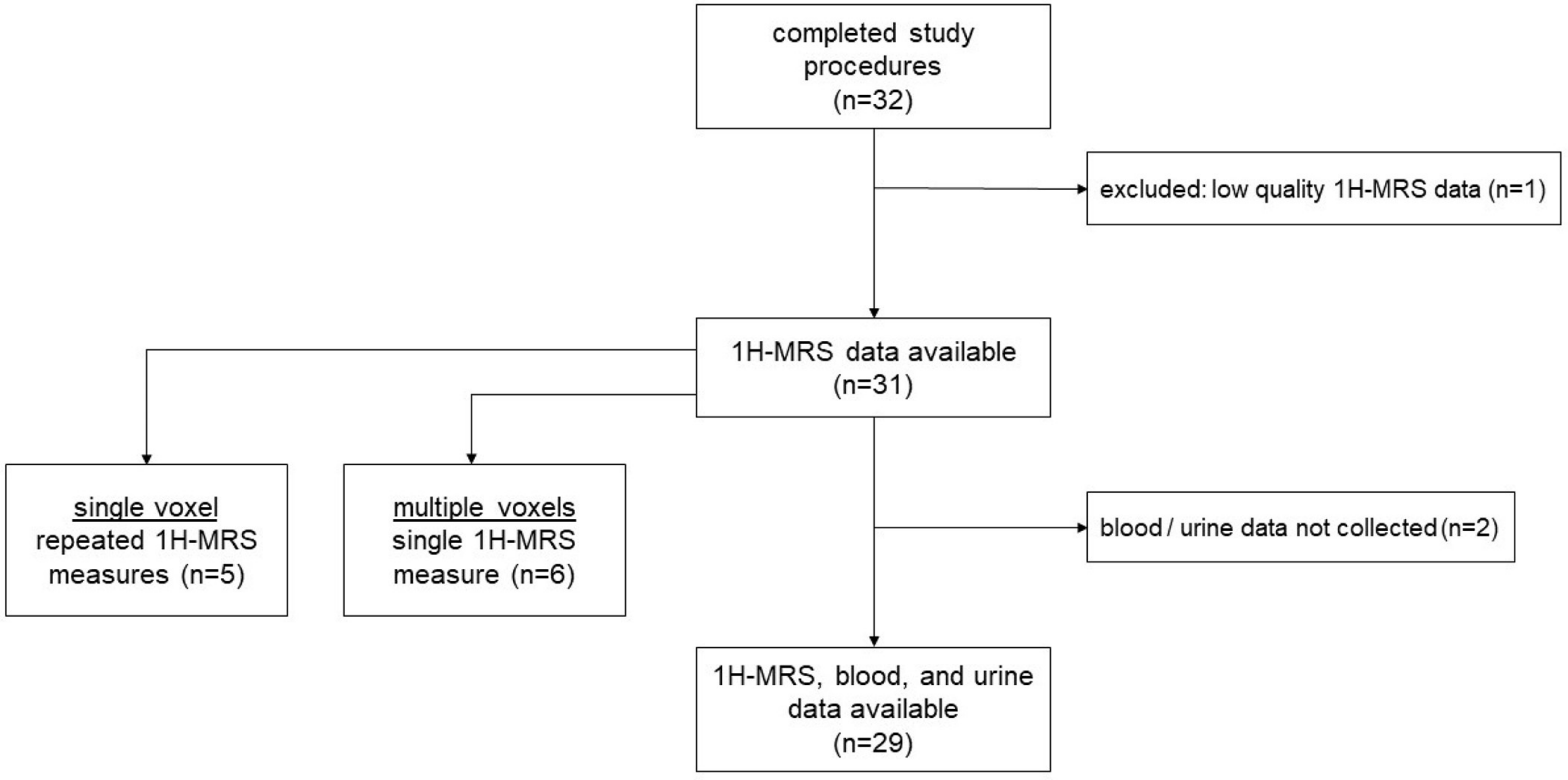
Flow diagram. Thirty-two participants completed study procedures, of which 31 had 1H-MRS data available, and 29 had 1H-MRS, blood, and urine data available.

Study recruitment is outlined in the flow diagram (Fig 2). Of the 32 participants who completed the study, one was excluded due to low quality of the 1H-MRS scans. Moreover, for two participants (one male and one female), 1H-MRS scans were performed, but blood and urine samples were not collected. These two participants were not excluded from the final study dataset, but only their 1H-MRS data were utilized for analysis. Thus, the final dataset included 31 participants, of which 15 (48%) were male, 25 (81%) were white, and 2 (7%) were vegetarians, while the average age of the study cohort was 38 ± 13 years (Table 1). Data from the two participants who maintained a vegetarian diet did not exhibit extreme outliers for any of the study testing modalities and hence were not removed from the final analysis. Thus, 1H-MRS data were available for all 31 participants, of which blood and urine data were available in 29 participants.

**Table 1:** Cohort characteristics.

Characteristic	Total (N = 31)
Age (Mean ± SD)	38 ± 13
Sex, Male (%)	15 (48)
Race, White (%)	25 (81)
Diet, Vegetarian (%)	2 (7)

### Sex-dependence of carnosine levels

The distribution of urinary (p = 0.33) and erythrocyte (p = 0.59) carnosine levels were similar between males and females (S1A and S1B Fig). However, muscle carnosine levels as determined from the C2 and C4 peaks were significantly higher in males as compared to females (S1C and S1D Fig).

### Urinary versus erythrocyte carnosine levels

**Fig 3:**
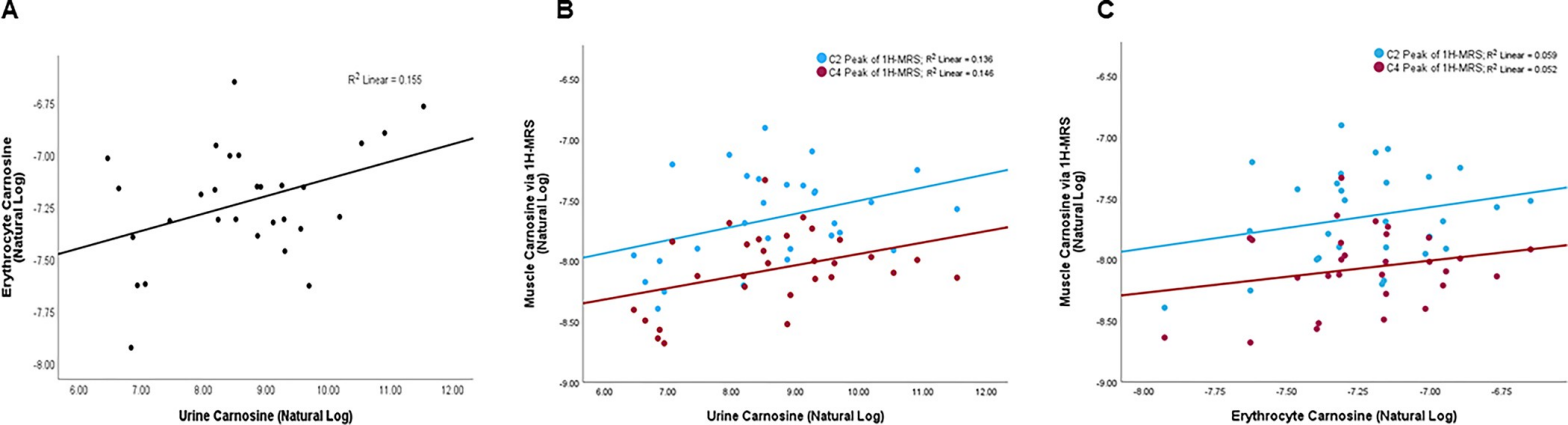
Unadjusted associations of carnosine levels. Illustrated is the: (A) analysis of carnosine as measured in the urine (abscissa) and in erythrocytes (ordinate) (R2 = 0.155; p = 0.035), (B) the analysis of carnosine as measured in the urine (abscissa) and in skeletal muscle (ordinate); both the C2 (blue: R2 = 0.136; p = 0.049) and C4 (red: R2 = 0.146; p = 0.041) MRS peaks were used, and (C) the analysis of carnosine as measured in erythrocytes (abscissa) and in skeletal muscle (ordinate); both the C2 (blue: R2 = 0.059; p = 0.20) and C4 (red: R2 = 0.052; p = 0.24) MRS peaks were used. Values are presented as log of absolute concentrations (nmol/mg creatinine or nmol/mg protein).

**Fig 4:**
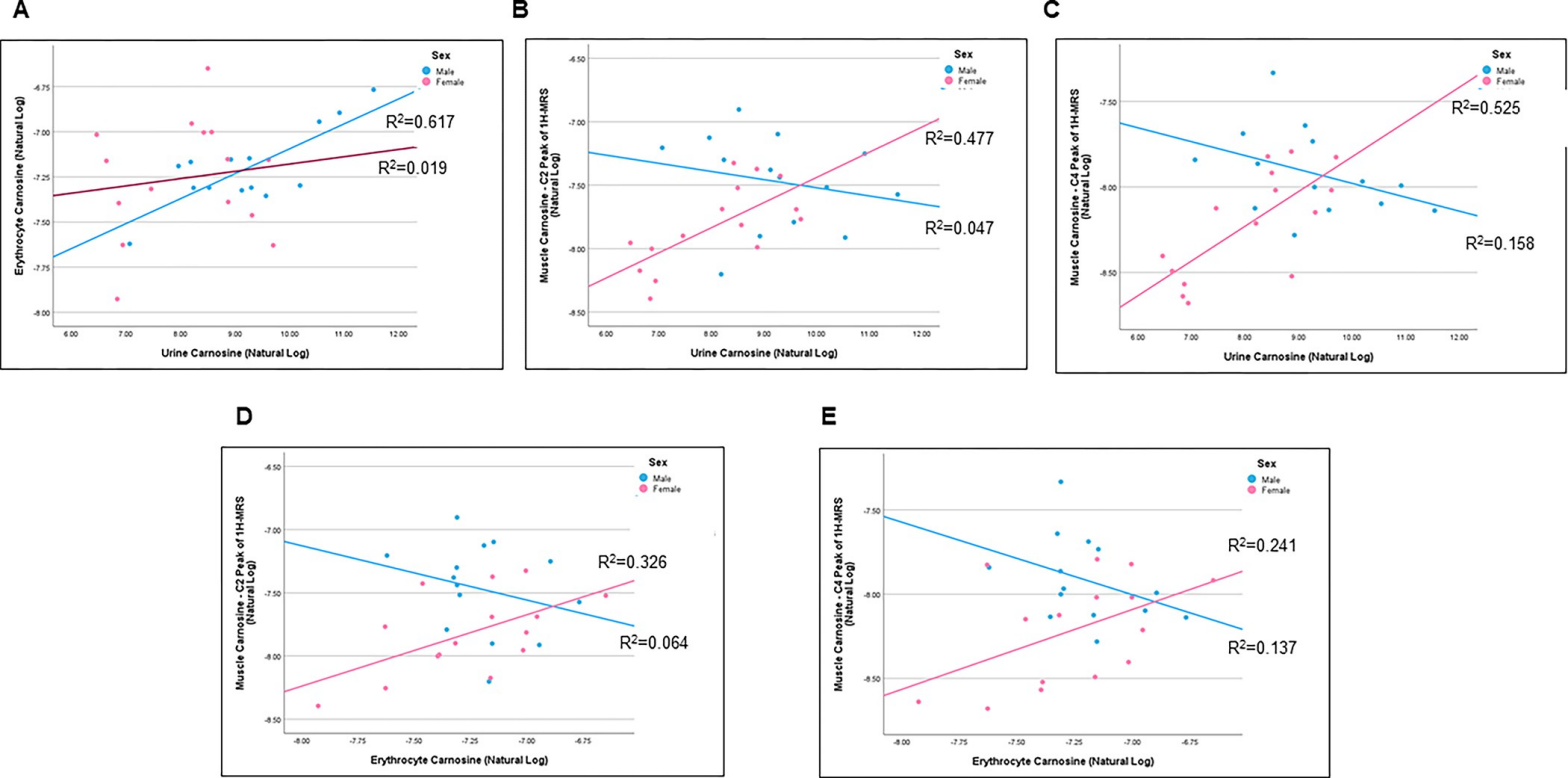
Sex-dependent relationships of carnosine in muscle, urine, and blood. Illustrated are the sex-specific relationships between carnosine in erythrocytes versus urine (A; male R^2^ = 0.617, female R^2^ = 0.019), the C2 peak of 1H-MRS versus urine (B; male R^2^ = 0.047, female R^2^ = 0.477), the C4 peak of 1H-MRS in versus urine (C; male R^2^ = 0.158, female R^2^ = 0.525), the C2 peak of 1H-MRS versus erythrocytes (D; male R^2^ = 0.064, female R^2^ = 0.326), and the C4 peak of 1H-MRS versus erythrocytes (E; male R^2^ = 0.137, female R^2^ = 0.241). Both the male (blue) and female (pink) sexes are shown distinctly.

Unadjusted levels of urinary and erythrocyte carnosine showed a positive linear correlation (R^2^ = 0.155, p = 0.035) (Fig 3A). Multivariate linear regression analysis of all study participants adjusted for age, sex, race, and diet also showed a positive association between urinary and erythrocyte carnosine levels (β = 0.16; 95% CI: 0.05, 0.27; p = 0.004) (Table 2). Sex did not show a statistically significant interaction/modification in the relationship between urinary versus erythrocyte carnosine (p = 0.34) in an adjusted generalized linear model (GLM). This is reflected in the distinct, but similar direction of the sex-stratified scatterplot representation of carnosine levels in these depots (Fig 4A).The association between urinary and erythrocyte levels in an adjusted GLM was positive among both males (β = 0.11; p = 0.044) and females (β = 0.22; P = 0.01) (Table 2).

**Table 2:** Regression analysis of measured carnosine levels.

Outcomes/Depots	Referent	β	95% CI	P Value
**All Participants (N = 29)**				
Erythrocyte Carnosine*	Urine Carnosine	0.16	0.05, 0.27	**0.004**
1H-MRS–C2 Peak*	Urine Carnosine	0.02	-0.13, 0.17	0.80
1H-MRS–C4 Peak*	Urine Carnosine	0.08	-0.05, 0.21	0.23
1H-MRS–C2 Peak^+^	Erythrocyte Carnosine	0.24	-0.19, 0.67	0.27
1H-MRS–C4 Peak^+^	Erythrocyte Carnosine	0.22	-0.16, 0.60	0.25
**Analysis Stratified by Sex**				
**Males (n = 14)**				
Erythrocyte Carnosine*	Urine Carnosine	0.11	0.003, 0.21	**0.044**
1H-MRS–C2 Peak*	Urine Carnosine	-0.20	-0.45, 0.05	0.12
1H-MRS–C4 Peak*	Urine Carnosine	-0.11	-0.28, 0.06	0.21
1H-MRS–C2 Peak^+^	Erythrocyte Carnosine	-0.52	-1.34, 0.31	0.22
1H-MRS–C4 Peak^+^	Erythrocyte Carnosine	-0.40	-0.96, 0.15	0.16
**Females (n = 15)**				
Erythrocyte Carnosine*	Urine Carnosine	0.22	0.05, 0.4	**0.010**
1H-MRS–C2 Peak*	Urine Carnosine	0.23	0.09, 0.36	**0.001**
1H-MRS–C4 Peak*	Urine Carnosine	0.32	0.23, 0.42	**<0.001**
1H-MRS–C2 Peak^+^	Erythrocyte Carnosine	0.55	0.15, 0.95	**0.007**
1H-MRS–C4 Peak^+^	Erythrocyte Carnosine	0.49	0.04, 0.94	**0.032**

### Urinary versus muscle (1H-MRS) carnosine levels

Unadjusted levels of urinary carnosine also showed positive correlations with the C2 (R^2^ = 0.136, p = 0.049) and C4 (R^2^ = 0.146, p = 0.041) peaks of 1H-MRS (muscle carnosine) (Fig 3B). However, adjusted multivariate linear regression analysis of all study participants did not show any significant association between urine levels versus the C2 (p = 0.80) or C4 peaks (p = 0.23) (Table 2).

A sex-stratified scatterplot of unadjusted values shows opposite directions of correlations between urinary carnosine and the C2 and C4 peaks of carnosine (Fig 4B and 4C) among males (negative correlation) and females (positive correlation). Sex-stratified analysis of an adjusted GLM in females showed a positive association between urine versus C2 peak (β = 0.23; p = 0.001) and C4 peaks (β = 0.32; p<0.001) (Table 2). However, among males, no statistically significant association was found between urine versus C2 peak (p = 0.12) or C4 peak (p = 0.21).

### Erythrocyte versus muscle (1H-MRS) carnosine levels

No statistically significant correlation was observed between unadjusted levels of erythrocyte versus C2 (p = 0.20) or C4 (p = 0.24) peaks of 1H-MRS (muscle) carnosine (Fig 3C). Similarly, when using an adjusted GLM, we did not observe any association between erythrocyte versus C2 (p = 0.27) or C4 (p = 0.25) peaks (Table 2).

Importantly sex showed an interaction/modification for the relationship between erythrocyte versus C2 peak (p = 0.019) and C4 peak (p = 0.017) carnosine in a GLM adjusted for age, sex, race, and diet. A sex-stratified scatterplot of unadjusted values shows opposite directions of correlations between erythrocytes versus C2 and C4 peaks of carnosine among males (negative correlation) and females (positive correlation) (Fig 4D and 4E). A sex-stratified analysis of an adjusted GLM in females showed a positive association between erythrocyte versus C2 peak (β = 0.55; p = 0.007) and C4 peaks (β = 0.49; p = 0.032) (Table 2). However, among males, no statistically significant association was found between urine versus C2 peak (p = 0.22) or C4 peak (p = 0.16).

### Reproducibility of MRS measures

To check the reproducibility of MRS measures in our system, we obtained repeat values of the C2 and C4 peaks in a single gastrocnemius voxel in a sub-set of five participants (Table 3). The coefficient of variance (CV) for multiple readings at single location for the C2 peak ranged from 2.9% through 11.7%, with a median of 7.5%. For the C4 peak, the CV ranged from 2.4% through 35.5%, with a median of 15.1%. Using another sub-set of six participants, we quantified the C2 and C4 peaks at multiple sites within the gastrocnemius of each participant (Table 3). The CV for single readings at multiple sites for the C2 peak ranged from 5.2% to 21.2% with a median of 13.0%. For the C4 peak the CV ranged from 8.0% to 21.3% with a median of 14.0%.

**Table 3:** Reproducibility of MRS measures.

Repeated measures in a single voxel		
Measure	COV (median)	COV (range)
C2 peak (all; n-5)	7.5%	2.9–11.7%
C2 peak (males; n = 4)	6.4%	2.9–9.0%
C2 peak (females; n = 1)	NA	NA
C4 peak (all; n = 5)	15.1%	2.4–35.5%
C4 peak (males; n = 4)	12.3%	2.4–21.5%
C4 peak (females; n = 1)	NA	NA
**Single measure at multiple voxels**		
C2 peak (all; n = 6)	13.0%	5.2–21.5%
C2 peak (males; n = 4)	12.2%	5.2–21.5%
C2 peak (females; n = 2)	14.6%	9.5–19.8%
C4 peak (all; n = 6)	14.0%	8.0–21.3%
C4 peak (males; n = 4)	14.4%	8.0–21.3%
C4 peak (females; n = 2)	13.1%	12.4–13.8%

## Discussion

The histidyl dipeptide carnosine plays an important role in physiological homeostasis. In this observational human study, we found that carnosine levels in erythrocytes were positively associated with urinary levels among all study participants and not modified by sex. In contrast, the association between levels of carnosine in urine versus skeletal muscle, or erythrocytes versus skeletal muscles was modified by sex. Therefore, muscle carnosine levels, as obtained by 1H-MRS, did not show a consistent association with urine or erythrocyte levels. Furthermore, the 1H-MRS method of carnosine quantification was highly sensitive to the location of measurement and not consistently reproducible. This study suggests that levels of erythrocyte carnosine may be reflective of that in other tissues. Furthermore, erythrocyte measures are likely not influenced by sex in contrast to measures in the skeletal muscle, where abundance and type may vary with sex and ethnicity. Thus, we suggest that the use of erythrocytes measures may be a viable alternative for invasive procedures like biopsy to determine endogenous levels of carnosine. The development of such non-invasive techniques will be of high utility in clinical studies.

Each of the measurement modalities we examined and the physiological depots that they represent has its unique importance in reporting on carnosine physiology. Each has their own limitations as well. Carnosine is most abundant in skeletal muscle and in theory, its measurement in this tissue by 1H-MRS gives an indication of steady state levels. However, there are some concerns about the widespread utility of this technique in clinical studies [33]. In addition to the necessity of having the proper instrumentation and trained staff for its operation, the procedure is time consuming, costly, and somewhat dependent upon the participants’ leg movements during a given scan. Therefore, the accuracy and reproducibility of data captured via 1H-MRS is dependent upon factors vulnerable to high variability. The low reproducibility of 1H-MRS-based measures reported in our study confirms these concerns and limitations. Problems in accurate quantification also arise because of the weak signal to noise ratio of the C2 and C4 MRS peaks as the amplitude of these signals are inherently low (Fig 2). Signal to noise ratio may also be dependent upon individual muscle characteristics such as fat content and the heterogeneous distribution of carnosine among different muscle fiber types. Thus, measured carnosine levels may be highly dependent upon voxel location as has been reported [33] and as we have observed in the current study, which may explain its lack of collinearity with erythrocyte or urine measures. The influence of such intra-operator variability is concerning, which may be exacerbated in multiple operator setups. Carnosine-specific signals may also be masked by competing signals from other imidazole-containing molecules. Moreover, Lievens et al. have recently reported an approximate 20% higher concentration of carnosine in muscles among males, as compared to females when measured via 1H-MRS in either gastrocnemius or soleus muscles [44]. While the current study also found a similar trend, studies with an appropriate power and sample size are required to further study this phenomenon. In addition to 1H-MRS, skeletal muscle carnosine can be measured in tissue collected by biopsy using mass spectroscopy. However, muscle biopsies are invasive and carry some significant risks and may be impractical for large-scale studies.

Whole blood samples represent another measurable source as there are carnosine stores in erythrocytes, which have proven useful in reporting on antioxidant potential [39]. While minimal amounts of whole blood are needed for these analyses, phlebotomy is associated with a small, though still viable risk of syncope. There may also be problems in the acquisition of the blood sample itself, such as difficulty in identifying useable vasculature, which we encountered in the current study. The overall utility of blood or erythrocyte carnosine measures may also be limited. Given rapid adsorption of nutrients from the GI tract into circulation, these measures may be mostly reflective of the most recent consumption of meat or other carnosine-rich food. Furthermore, the measurement of carnosine in blood plasma is complicated and challenging, given its rapid hydrolysis into histidine and β-alanine by carnosinase, which is abundant in circulation [2].

Finally, the measurement of carnosine in urine samples has found increasing acceptance [24, 25, 34]. One of the reasons for this is that urine sample collection and analysis is fairly convenient and feasible using standardized methods. The measurement of carnosine by mass spectroscopy in urine or blood samples has standardized processes with fewer limitations which are routinely used in most laboratories [25, 39]. The collection of urine samples is quick, easy, and adequate volumes are easily obtained. Although additional sample preparation is required, these procedures are quite manageable within realm of standard laboratory procedures. There is only a need for adequate analytical instrumentation and personnel trained for its use. Moreover, beyond levels of native carnosine, urine samples can be used to quantify levels of carnosine-adducts, enabling indirect assessments of individual xenobiotic exposure [25] and disease risk [24].

**Fig 5:**
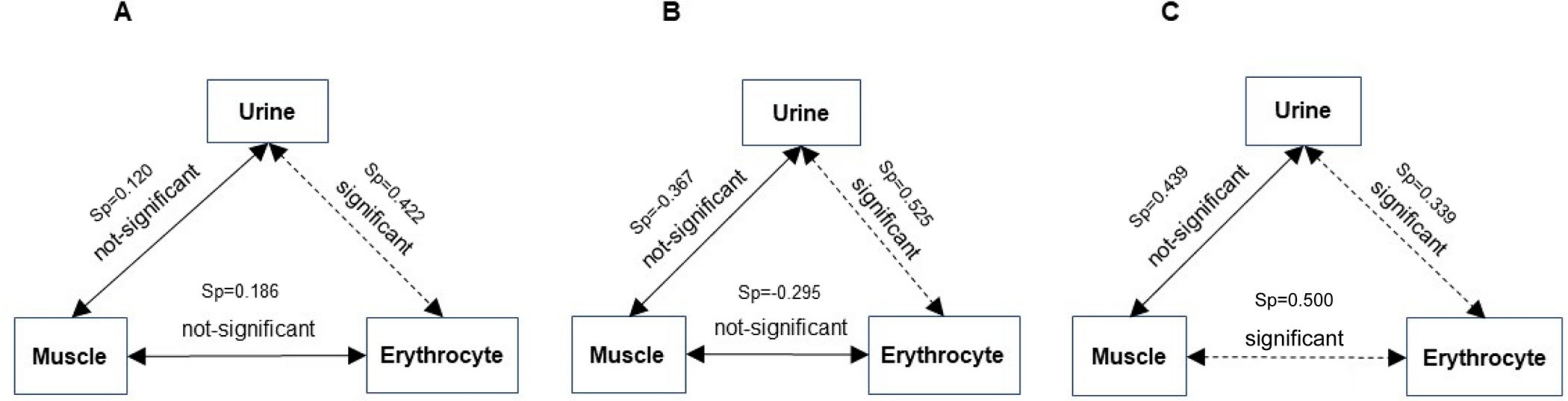
Relationship between carnosine in three *in-vivo* depots. Illustrated are the relationships in carnosine levels among all participants (A: n = 29), in males only (B: n = 14), and in females only (C: n = 15) using generalized linear models adjusted for age, sex, race, diet, and creatinine. Statistical significance is depicted with dotted lines indicating a significant two-tail positive association and solid lines indicating non-significant associations. Spearman correlation coefficients (Sp) are also listed for each relationship.

In conclusion, we provide the first-ever comparison of endogenous carnosine levels in three distinct depots in humans (Fig 5). We found that urinary and erythrocyte carnosine levels displayed a positive relationship in both sexes, whereas the relationships between erythrocyte and muscle levels, and urinary and muscle levels, were sex-dependent (Fig 4). Thus, the relationship between urinary and erythrocyte carnosine levels may be stable and not influenced by confounders. In contrast, those levels determined from muscle scans may be influenced by one or more confounders. One limitation of the current study is that absolute levels of skeletal muscle carnosine were not determined in biopsy samples, as we were not able to recruit participants for this analysis. Nevertheless, we would like to suggest the widespread suitability of urinary and erythrocyte measurements, which may be viable, non-invasive alternatives for determining levels of carnosine or related analytes. Development of such techniques will be especially useful for large-scale clinical studies, especially those which assess levels before and after interventional approaches.

## Supporting information

S1 Fig Sex-dependent comparison of carnosine levels in muscle, urine, and blood. Illustrated is the difference in carnosine levels between males (blue) and females (red) in urine (A), erythrocytes (B), the C2 peak of 1H-MRS (C), and the C4 peak of 1H-MRS (D), using non-parametric Mann-Whitney U tests. Values for urinary carnosine are in nmol/mg creatinine while those for erythrocyte carnosine are in nmol/mg protein.
(PDF)
