# Supplementary material for: Comparative evaluation of different modalities for measuring in vivo carnosine levels
Source: PLoS One. 2024 Mar 27;19(3):e0299872. doi: 10.1371/journal.pone.0299872 (PMC10971688; doi:10.1371/journal.pone.0299872)
Supplement: S1 Fig — Illustrated is the difference in carnosine levels between males (blue) and females (red) in urine (A), erythrocytes (B), the C2 peak of 1H-MRS (C), and the C4 peak of 1H-MRS (D), using non-parametric Mann-Whitney U tests. Values for urinary carnosine are in nmol/mg creatinine while those for erythrocyte carnosine are in nmol/mg protein. (PDF) [file pone.0299872.s001.pdf]

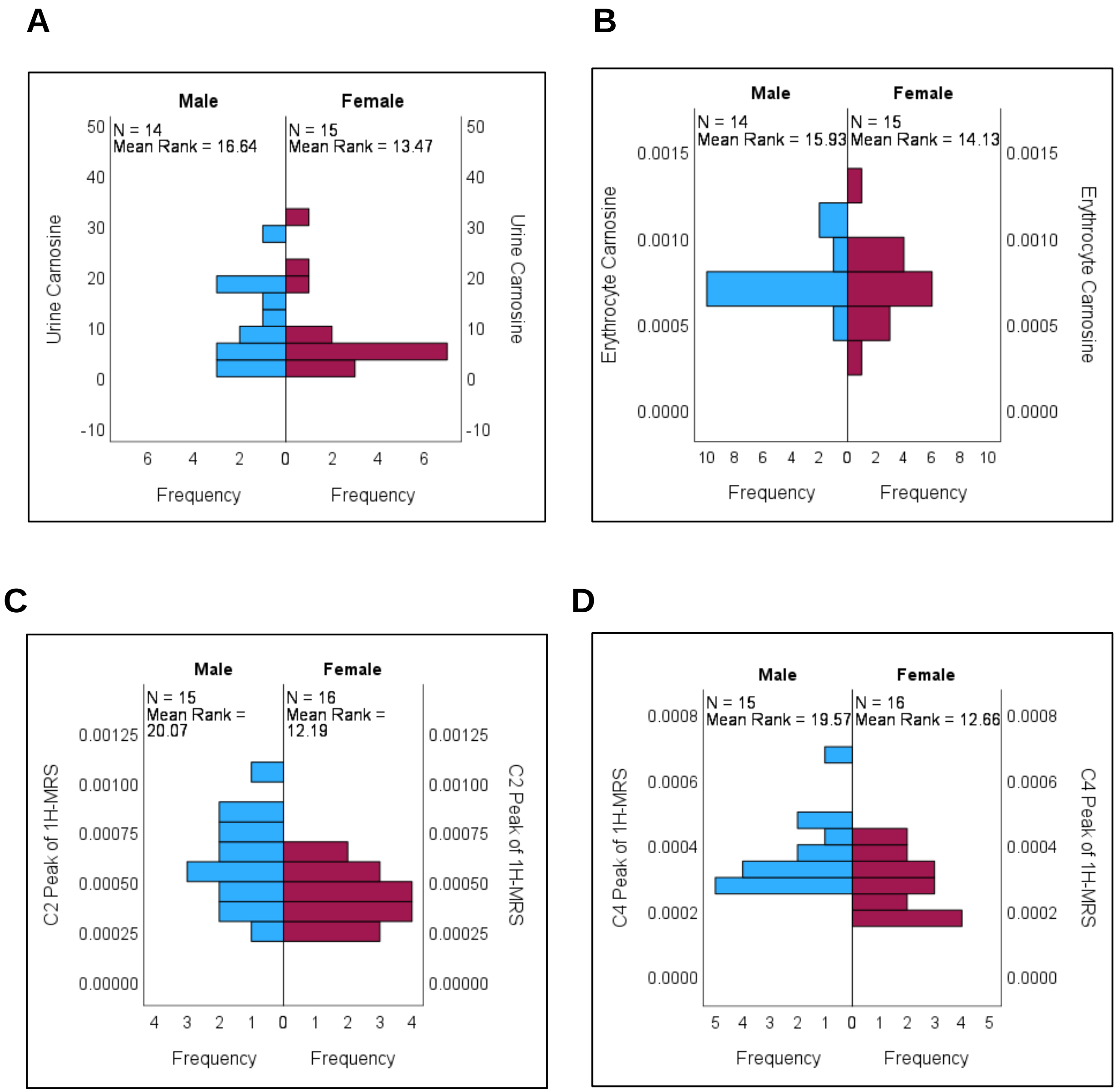

Supplementary Figure 1. Sex-dependent comparison of carnosine levels in muscle, urine, and blood. Illustrated is the difference in carnosine levels between males (blue) and females (red) in urine (A), erythrocytes (B), the C2 peak of 1H-MRS (C), and the C4 peak of 1H-MRS (D), using non-parametric Mann-Whitney U tests. Values for urinary carnosine are in nmol/mg creatinine while those for erythrocyte carnosine are in nmol/mg protein. Values for urinary carnosine are in nmol/mg creatinine while those for erythrocyte carnosine are in nmol/mg protein.
